# Differences Help Recognition: A Probabilistic Interpretation

**DOI:** 10.1371/journal.pone.0063385

**Published:** 2013-06-03

**Authors:** Yue Deng, Yanyu Zhao, Yebin Liu, Qionghai Dai

**Affiliations:** Department of Automation, Tsinghua National Laboratory of Information Science and Technology (TNList), Tsinghua University, Beijing, China; University of Adelaide, Australia

## Abstract

This paper presents a computational model to address one prominent psychological behavior of human beings to recognize images. The basic pursuit of our method can be concluded as that differences among multiple images help visual recognition. Generally speaking, we propose a statistical framework to distinguish what kind of image features capture sufficient category information and what kind of image features are common ones shared in multiple classes. Mathematically, the whole formulation is subject to a generative probabilistic model. Meanwhile, a discriminative functionality is incorporated into the model to interpret the differences among all kinds of images. The whole Bayesian formulation is solved in an Expectation-Maximization paradigm. After finding those discriminative patterns among different images, we design an image categorization algorithm to interpret how these differences help visual recognition within the bag-of-feature framework. The proposed method is verified on a variety of image categorization tasks including outdoor scene images, indoor scene images as well as the airborne SAR images from different perspectives.

## Introduction

Visual information understanding is a long-standing topic that has been extensively discussed in both the communities of vision research and artificial intelligence. Over last decades, a large number of prominent works have been devoted to this challenging task from diverse ranges of perspectives including biological investigation [1]–[3], psychological research [4]–[6] and computational methods [7]–[10]. In this paper, we propose a computational approach to reveal the differences of various images and will interpret how the differences help computers to conduct visual recognition tasks.

The fundamental observation that inspires our work is the behavior of human beings to distinguish different images. Psychologically, when distinguishing two images, we pay much attention to their differences rather than the common characters. For example, when categorizing two images from bedroom category and office category, the most representative information that helps recognition are the “beds” and “computers”. When finding the patterns for beds, it is easy to tell that the image is from the bedroom. Similarly, when finding the features of a computer in an image, it probably describes an office scenario. However, in both of these two images, other common patterns are also collected, *e.g.* the wall and the ground. These common features help less to distinguish these two images because they appear in both of them.

Such desired information selection mechanism is easily implemented as visual information processing by human brain. Unfortunately, it can hardly be addressed by computers. Therefore, to fill the gap, we propose a probabilistic computational framework to model such behavior by statistical machine learning [11]. To better describe the computational model, we clarify two important concepts in statistics, *i.e.* generative model and discriminative model [12].

Loosely speaking, generative models, *e.g.* Gaussian Mixture Models (GMM) and the topic model, play the role of interpreting how the observations are generated/sampled from a probabilistic distribution. They are different from discriminative models in that generative models learn a joint probability distribution of both the observations and the corresponding labels. Generative models are used in machine learning for either modeling data directly (*i.e.* modeling observations drawn from a probability density function), or an intermediate step of forming a conditional probability density function.

In contrast, discriminative model generally reveals what makes data different among classes. In machine learning, discriminative model is always described by a conditional probability distribution which expresses the dependence of the labels on the observed features. Discriminative models differ from generative models in that they do not allow one to generate samples from the joint distribution of labels and features. However, for tasks such as classification, they do not require the joint distribution and, in most cases, can yield superior performance. Widely used discriminative models in machine learning include the Support Vector Machine (SVM), Multinomial Logistic Regression (MNL) [12] and Conditional Random Fields (CRF) [13].

In our work, a generative computational model is developed to describe how the image features in different classes are generated by a joint probability density function (pdf) of features and their labels. In addition, a discriminative model is incorporated into the Bayesian model to reveal the discriminative patterns among different classes. The whole model is solved in an Expectation-Maximization manner for parameter estimation and latent variables inference.

Using Bayesian model and probabilistic approaches for visual information understanding has been extensively discussed in communities of computer vision and machine learning. In [14], a Bayesian model is used for pattern detection, *e.g.* circle object, on images. However, their approach shed no light on the discriminative aspect. Probabilistic inspired metric [15], [16] has also been incorporated into typical subspace models for visual feature extraction. But these approaches do not explicitly enhance the discriminative property of the extracted features. Different from existing works on image feature extraction, the model discussed in this paper is fully conducted in a Bayesian paradigm. Thanks to the flexibility of probabilistic models, the features selected from our model exactly reveal the discriminative information of an image in a much detailed manner.

In previous works, discriminative methods have been exploited into typical image coding framework to improve the image categorization performances. In [17] and [18], the discriminative functionality has been incorporated into the sparse coding method to learn the discriminative dictionary. However, the sparse coding methods pay too many computational costs to learning the dictionary and encoding one image, which requires solving the 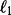 type optimization for multiple times. In [19], a probabilistic based discriminative kernel has been defined to evaluate the distances of two images. It shed light on discriminative learning and could achieve very good performances by enhancing image differences. However, the work [19] only implicitly exploits the image differences and could not explicitly indicate what kind of features on the image are discriminative features. In our approach, thanks to the generative framework, we could exactly tell what kind of features cause the differences among images. Moreover, we will introduce how to construct the discriminative codebook by utilizing these image differences. Then, image encoding may follow the efficient paradigm of soft assignment [20].

To verify the effectiveness of the proposed framework, we apply the model on a number of image categorization tasks including natural scene recognition [21], earth observation (airborne images) recognition and indoor scene categorization [22]. Meanwhile, the experiment is conducted on bi-class classification and multi-class scene categorization tasks. It is glad to see from the bi-class categorization task that with the proposed framework, we can even accomplish the categorization work without training extra classifiers. The features learned by our model naturally exhibit significant discriminant structures for classification.

## Materials and Methods

In this part, we describe our probabilistic model that interprets how the contents of different images are “generated”. To describe the image contents, we follow the bag-of-feature method [23] to extract the image features at the pixel-level. In detail, a grid-based method is used to extract the dense SIFT features [24] from the images. The SIFT features are extracted on 16×16 pixel patches sampled every 8 pixels. For the ease of presentation, we define different image categories as 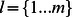, which represents 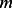 classes. After extracting features from all the images, 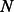 feature-and-label pairs, *i.e.* 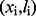 are obtained, where 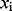 records the SIFT feature and 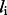 denotes the label of a certain feature.

### Method

In this paper, we consider local image features as two types: discriminative features and common features. The discriminative features are desired for classification purpose which significantly represent the class attributes. On the contrary, the common features appear in different categories and do not imply significant label information. To simultaneously model the generation of feature-and-label pairs, a probabilistic model is defined as
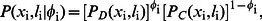
(1)where 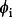 is the latent variable which equals to one if the 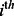 feature is a discriminative feature and zero if the feature belongs to the common feature set. In (1), 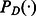 and 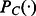 are used to represent the joint pdf of the feature-and-label pairs of the discriminative part and common part, respectively. Meanwhile, the probabilistic generative model for 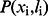 is subject to the following expression,



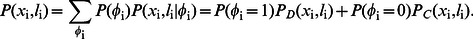
(2)To mathematically describe the joint pdf of discriminative features and their labels, *i.e.* 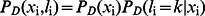, the conditional probability 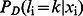 should be explicitly defined. These discriminative features are class-specific and are different between categories. Therefore, this kind of features imply prominent label information and, mathematically, can be well explained by a discriminative functionality. In this paper, we exploit the Multinomial Logistic Regression (MNL) to model the conditional probability 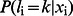 due to the following two reasons.

First, an MNL explicitly minimizes the logistic empirical losses as the objective whose performances have been widely admitted in a number of practical applications [25], [26]. As indicated in [12], the accuracy of MNL is similar to support vector machine (SVM) in addressing many real-world tasks. Meanwhile, the remarkable advantage of MNL for our case is its explicit probabilistic definition and its ease of being incorporated into our probabilistic model seamlessly. Accordingly, we define the pdf for the discriminative part as,
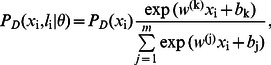
(3)where 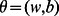 is the parameter of the MNL. Besides, a Gaussian mixture model (GMM) is adopted to encode the prior of 
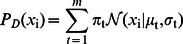
, where 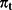 is the mixture coefficient and 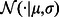 is the Gaussian distribution with 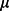 and 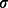 as the mean and the standard deviation, respectively. In this paper, we select the number of mixture components as 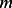 which corresponds to the number of classes.

Similarly, it is easy to model the common feature generation part as,
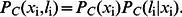
(4)


**Figure 1:**
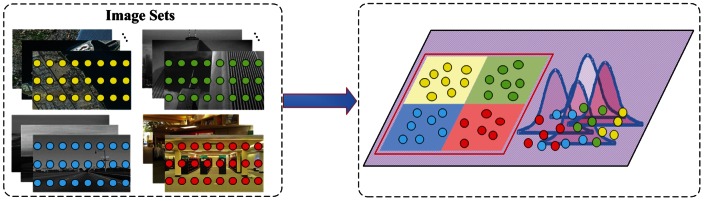
An overview of the proposed algorithm. In the figure, the round dots represent the extracted image features (local SIFT features) and different colors represent different category attributes of the image features. In the left panel, the local image features are extracted from all the images in multiple classes at the pixel-level. In the right panel, the proposed algorithm automatically determines what kind of local features represent the differences between classes and what kind of features are common ones in multiple classes. The features in the red rectangular are the discriminative features which are well modeled by a Multinomial Logistic Regression model. Besides, the common features in multiple classes do not take significant class information and are modeled by a Gaussian Mixture model.

In the above equation a GMM model is used to model the prior of 
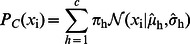
, where 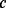 is the number of components selected in the model. Besides, it is interesting to note that (4) describes the common features in multiple classes, therefore, the feature does not exhibit significant category attribute. Mathematically, the feature is independent to the label and thus 
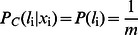
, where 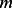 is the number of classes. Till now, we have explained both the discriminative part and the common part of the model. An overview of the proposed method is provided in Fig. 1.

Here, we remark on the parameter set 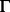 used in the model. 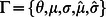, where 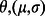 are the parameters for the logistic regression and GMM in (3), respectively. 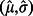 is the parameter of the GMM in (4). In statistics, to estimate the parameters, a maximum likelihood (ML) formulation should be maximized,
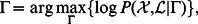
(5)where 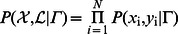. Unfortunately, the above log-likelihood is not solved analytically due to the incorporation of the latent variable 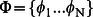. In this paper, we use the Expectation-Maximization (EM) algorithm to efficiently solve the model.

### An EM solution

EM method is widely used in statistics to solve probabilistic models with latent variables [27], [28]. In [29], it is proven that EM method establishes the lower bound of the likelihood in (5) using the Jensen's inequality. Then, the lower bound is iteratively maximized and the whole optimization is solved by alternating between two steps, *i.e.* E-step and the M-step.

- In the E-step, the conditional expectation for the complete data likelihood is determined with the current estimation of parameters.
- In the M-step, the parameter is updated by maximizing the conditional expectation in the E-step.

The EM algorithm is a special case of the more general Majorization Minimization (MM) algorithm [30]. In our model, we first calculate the conditional expectation for the complete-data likelihood [31],
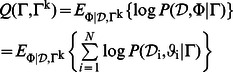
(6)


Here, for the ease of illustration, we define the data set as 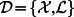. Then, in the M-step, the Q-function is maximized to get the new parameter, *i.e.* 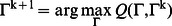. The E-step and the M-step are iteratively processed until the convergence is reached. In our simulation, the convergence criteria is regarded as satisfied when the change of the objective between two iterations is below 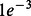.

In our model, one critical step is to estimate conditional probability for the latent variables given the model parameters. We define 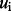 and 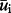 as follows,
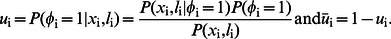
(7)


Physically, 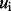 represents the probability that the 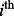 feature is a discriminative feature and, on the contrary, 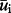 records the probability that the 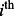 feature belongs to the common feature set. Accordingly, we present the whole EM framework in Table 1. The detailed derivations for the EM algorithm and parameter updating rules are provided in File S1.

**Table 1:** The EM algorithm for model inference and learning.

**Input:** Feature points *X* = {*x*_1_,…*x*_N_} and their labels *L* = {*l*_1_…*l*_N_}
**Initialization:** 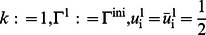 .
**Repeat**
** E-Step:**
Estimate 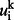 and 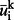 according to (7);
Calculate 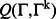 according to (6) with the current estimation of 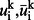 ;
** M-Step:**
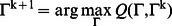 ;
*k* = *k*+1;
**Output:** 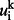 .

To solve the EM model in Table 1, the MNL in the discriminative part is initialized by fitting the original data with a logistics regression. In the other part of the model describing the common features, a Gaussian Mixture Model is applied to fit all the local image features, and then the obtained parameters are used for initialization.

### Feature Encoding by Exploiting Image Differences

In previous parts, we have stated how to determine the discriminative patterns from multiple images. However, the final goal for many scene recognition tasks is for categorization. The selected features are only local features (extracted at the pixel-level). Therefore, to capture the information of the whole image, some further coding steps are desired to generate features at the image level. In computer vision, one prevalent method for image coding is the bag-of-feature method in which all the local features on the images are assigned to a dictionary with multiple codewords. To make this paper self-contained, we briefly review the procedures of bag-of-feature method for image-level feature encoding.

As discussed previously, on one image, we can generate many local SIFT features. These local features cannot be directly used for image-level understanding. To describe an image, we should generate an image-level feature by exploiting the local descriptors on it. One prevalent approach to construct such an image-level feature is to construct a codebook and then assign local descriptors to the codebook, forming the image-level feature for classification.

Codebook construction is a training procedure, which clusters bags of local features, *e.g.* SIFT features, on the training images into 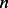 representative codewords. Then, during the second step, the features on the test images are assigned to their corresponding codewords in the codebook. An image-level feature, *i.e.* a histogram, is accordingly generated during codewords assignment to represent an image.

**Figure 2:**
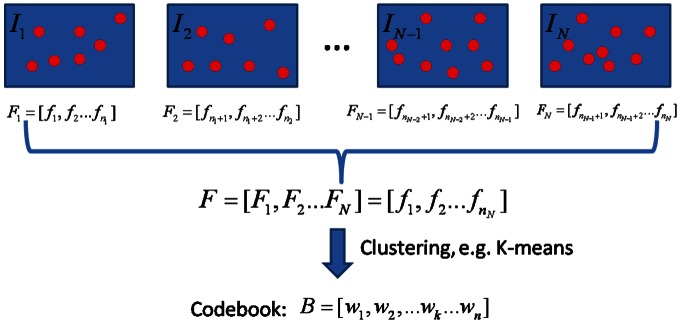
The generation of a codebook with 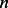 codewords. The blue rectangle means the training images and the red dots represent the local features on the image.

Fig. 2 summarizes the main procedures of codebook generation. Firstly, the local features in each image should be extracted and then thousands of local descriptors from multiple images are clustered into 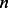 centers. These centers are the codewords in the codebook. Therefore, the codewords in the dictionary are not determined by any specific local feature. Instead, they capture the common properties of all the local features from training images. After constructing the codebook, the local image features are assigned to the codewords to form an image-level histogram. In this paper, we adopt the soft assignment method introduced in [20] to conduct codeword assignment.

From the above discussions, it is apparent that the codewords play very critical role in generating an image-level feature. Therefore, it is desired that the generated codewords could enhance the discriminative information of different images. Fortunately, the proposed Bayesian model has provided feature types (*i.e.* discriminative or common features) for all the local features. Accordingly, it is nontrivial to exploit such prior to generate more descriptive codewords for image categorization. In typical codewords generation, all the local features from the training images are used to generate the codewords by clustering. In this work, the codewords are only clustered by those discriminative local features selected by our algorithm. We regard 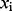 as a discriminative feature if 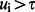 and 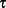 is fixed as 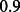 in our simulation. The selected discriminative features reveal the differences among multiple images. Therefore, when using these discriminative local features for clustering, the centers, *i.e.* the codewords, may better reveal the discriminative information on the images.

## Results

In this part, we experimentally verify the performance of the proposed statistical model on treating real world images. We will report some numerical properties of the statistical model and then two experiments on bi-class and multi-class classification will be respectively conducted to verify the effectiveness of the selected discriminative features for image categorization. But before the experimental discussions, we first state the datasets that are used in the experiments.

### Data Collection

The data are from three datasets, Synthetic Aperture Radar (SAR) dataset, Fifteen Scene dataset and MIT Indoor Scene dataset, among which SAR dataset is established by us.

SAR consists of six ground categories: city, countryside, mountain, river, seaside, and water area. Each category in SAR contains more than 50 images. Most of these scene images are captured in China with our own airborne SAR devices. The original SAR data are converted into gray scale images and the experiments are only performed on these pseudo-gray-scale images. These images are cropped from a large SAR map to around 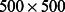 pixels.

The Fifteen Scene is a dataset of fifteen natural scene categories released by Fei-fei *et al* [21]. The image classes include bedroom, suburb scene, industrial, kitchen, living room, coast, forest, highway, inside city, mountain, open country, street, office, store, and tall building. Each category in Fifteen Scene dataset contains over 200 images. This dataset has been widely used in the field of computer vision as a benchmark dataset for scene categorization. In our experiment of bi-class recognition, most of the image classes are from the Fifteen Scene dataset.

In high level vision, indoor scene recognition is a challenging open problem. Most scene recognition models that perform well with respect to outdoor scenes work poorly, however, on indoor scene recognition. The MIT Indoor Scene dataset is released by Quattoni *et al.* in [22]. The dataset contains 67 indoor categories, and a total of 15620 images. The dataset contains indoor scene images ranging from airport inside, bookstore, library, mall, and video store to other scenes such as warehouse, dining room, fast food restaurant, and computer room. The number of images varies across categories, but there are at least 100 images for each category. This dataset has become a benchmark for indoor scene recognition.

### Numerical Performance

**Figure 3:**
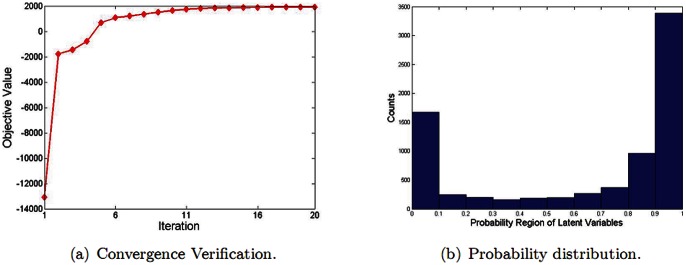
Numerical Verifications. (a) the objective value with the iterations and (b) the probability distribution of discriminative significance indicator 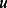.

For numerical analysis, we first report some results associated with the convergence of the learning model. As our statistical model is solved with an EM framework, we first show the objective value of the Q-function, *i.e.* Eq. (6), in Fig. 3 (a). From the result, it is obvious that the Q-objective function of EM algorithm increases along with the iterations. It is also apparent that the whole optimization framework reaches convergence within 20 iterations.

Besides, we also report the learning results of the whole EM algorithm. The basic pursuit of Table 1 is to find the features that capture significant label information of different classes. With this purpose, latent variables 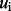 are introduced for each feature in the training process. According to Table 1, the discriminative saliency variables 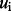 are the desired output of the probability model. If 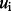 is large, the corresponding feature 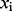 is probably a discriminative feature whereas a small 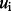 implies that 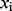 is a common feature shared by multiple classes.

**Figure 4:**
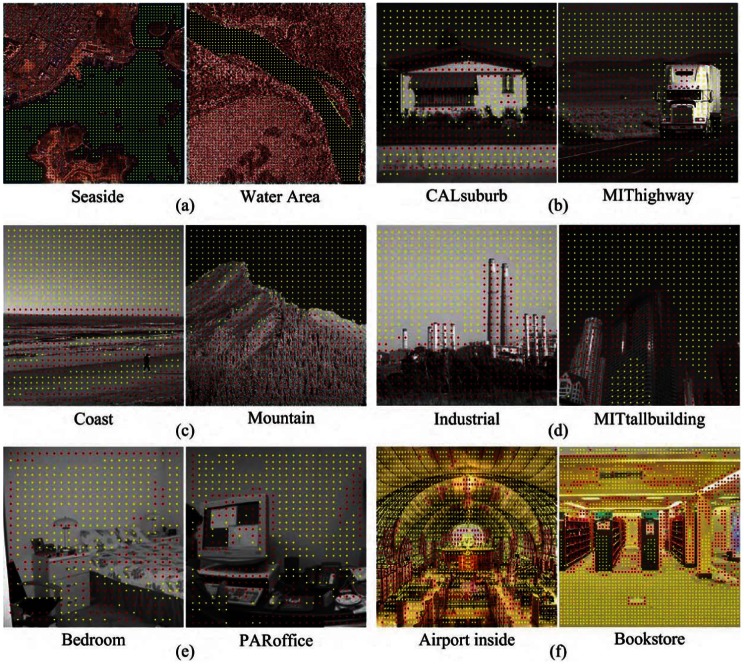
Sample images and discriminative feature selection results on bi-class categorization tasks. In each sub-figure, the red dots represent differences of the two classes and the yellow dots are their common contents.

To find the numerical property of the learning results, we conduct experiments on a bi-classes classification task. We apply our model to the Seaside and Water Area classification task of Fig. 4 (a). The statistical results of the distribution of 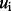 are reported in Fig. 3 (b) where the abscissa divides the probability of [0,1] into ten regions. The ordinate records how many local features in the training set fall into the corresponding region. From the results, it is obvious that most features are separated into two groups of discriminative features (with large 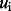) and common features (with small 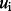), respectively. The latent variables larger than 0.9 indicate that their corresponding features are very likely to be discriminative ones and will probably help recognition. In contrast, those on the left with probability lower than 0.1 denote common features in both classes, whereas these features have little contribution to the recognition. The results also serve to verify that our statistical model could exactly distinguish these two kinds of features from practical images.

### Results on Bi-class Recognition

In this subsection, the proposed model is implemented on real image classification tasks. First, we consider performing the discriminative feature selection experiments on bi-class recognition cases.

Six pairs of classes that cover indoor, outdoor and aerial scenarios are tested in the experiment. For each pair of classes, our model tries to select out the common features as well as the features that are more distinguishable for classification (discriminative features). The sample images of each classification task and our feature selection results are provided in the sub-figures (a) to (f) in Fig. 4. In these images, the red dots represent the discriminative features (*i.e.* the differences) and the yellow dots are common features shared by two classes. Specifically, in our experiment, all features from images of each class are included and clustered into 4000 centers by K-means. Then, the clustered features (as many as 8000 for two classes) are included in the training process of the model. Discriminative and common clustered features will be accordingly determined by the estimation of their latent variables after the convergence of the EM algorithm.

We analyze the visual results in Fig. 4. In sub-figure (a), the images of Seaside class and Water Area class both have water regions. Therefore, the water regions are selected as common features and other contents are labeled as discriminative features. This observation is also found in Fig. 4 (b) to (d) that the sky in these images are selected as common features and other contents carrying significant category information are denoted as differences. In Fig. 4 (e), for the indoor scenario, the walls are regarded as common features while the bed in bedroom class and the computer in office class are selected as discriminative features. Finally, when distinguishing the images from airport class and bookstore class in Fig. 4 (f), the ground and the wall provide less category information. The discriminative patterns mainly distribute on the counter (in airport scenario) and books (in bookstore scenario).

The discussions aforementioned only provide intuitive results. More strictly, we will verify the effectiveness of discriminative feature selection (DFS) via quantitative analysis. The prominent contribution of this work is that we can identify the discriminative local features from common ones in multiple images. Therefore, by utilizing these discriminative features, a discriminative codebook (DC) is consequently constructed for image-level histogram generation. The DC is different from typical codebook (TC) that in DC the codewords are the centers of the discriminative local features after Bayesian learning. On the other hand, in typical codebook, the codewords are clustered by using all the local SIFT features from the training images.

**Figure 5:**
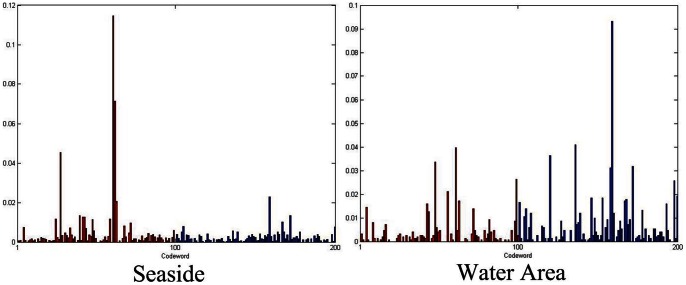
Histograms of two images in Fig.4 (a) generated using discriminative codewords. The red region represents the codewords from the seaside category and in the blue region are codewords from water area category.

For image classification, in typical BoF framework, the image features (obtained after image encoding) are fed into a discriminative classifier, *e.g.* a Support Vector Machine (SVM) for final categorization. However, in this bi-class categorization task, if the discriminative codebook are utilized, we can even classify the images just by their feature saliency without training an extra classifier. The basic idea for feature-saliency-based bi-class categorization algorithm can be well illustrated by the following toy demo. We generate the discriminative codebook for the bi-classes categorization task in Fig. 4(a). After generating the codebook by only using the discriminative features, we assign the images to the codewords according to [20] and the visual results are provided in Fig. 5. It is obvious that in the Seaside histogram, more SIFT features are assigned to the first 100 codeword. Note that these 100 codewords are generated by clustering all the discriminative features from Seaside determined by our model. Therefore, by simply calculating the energy distribution of the histogram, the image can be accordingly classified.

In the previous paragraph, we have introduced the feature-saliency-classifier (FSC) for bi-classes image classification by using discriminative codebook. Of course, the same features can also be classified following the routine way of training an SVM. We will experimentally compare these two classifiers with the same features in Table 2. Besides, we also report the classification results on the features generated by using typical codebook. In the typical codebook, the codewords no longer contain class information and thus we cannot classify the images by exploiting feature saliency. They can only be classified by training an SVM. In this simulation, 1000 codewords are selected in the codebook. For training, 10 images in the SAR dataset are randomly selected as training samples. For the remaining five tests on natural images, 50 images in each category are selected as training samples. In each task, the experiments are randomly repeated for 10 times and the average classification accuracy and their standard deviations are tabulated in Table 2.

**Table 2:** Recognition Accuracy of Bi-class Classification Tasks(%).

Classes	Seaside/ WaterArea	Bedroom/ PARoffice	CALsuburb/ MIThighway	Industrial/ MITtallbuilding	MITcoast/ MITmountain	Airport inside/ Bookstore
DC+FSC	90.3±0.7	83.4±0.8	**94.2**±0.7	87.8±0.6	88.1±0.8	73.7±0.6
DC+SVM	**91.8±0.6**	**89.5±0.5**	**93.4±0.6**	**88.1±0.7**	**91.5±0.5**	**76.4±0.6**
TC+SVM	88.41±0.5	85.1±0.7	90.3±0.5	85.5±0.7	89.3±0.7	72.9±0.7

In the Table, the first and the second row report the classification results by using the DFS-codebook. The first row shows the classification result with FSC, and the second row shows classification accuracy with SVM classifier. In these two rows, the same features are fed into two different classifiers, *i.e.* the FSC and SVM. From the result, obviously, SVM has higher performance than that of FSC in five out of six tasks. There is no surprise to see such improvements because SVM is a strong classifier that requires extra training costs. FSC only vote for the classification by counting the feature saliency and requires no training. By reporting the results of FSC in this part, we just want to claim that the features learned after DFS itself have exhibited significant class attributes and they can even be easily classified by a classier as simple as FSC. From the result, it is also interesting to note that for the same features with DFS-codebook, the performance of FSC is comparable to the results of SVM.

The third row in the table shows the classification results by using typical codebook, which means that the codebook is generated by simply clustering all the local features in the images. It is obvious that the codewords in this codebook have no discriminative characters. By comparing the classification results in the second and third rows, it is found that by using the same classifier, the performance of DFS-codebook is much better than the typical codebook. This finding serves to verify that utilizing the differences among images actually helps recognition.

### Results on Multi-class Categorization

In previous parts, the proposed algorithm is evaluated on bi-class classification task. In this part, we consider a more general case of applying the proposed model to improve the classification accuracy on multi-class tasks. For multi-class case, the learning procedures of discriminative feature selection are processed via a one vs. others paradigm. In a nutshell, we assume that there are 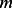 classes in total. To select the discriminative features for the 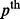 class, the features for the 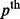 class are regarded as positive features and the features from other 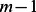 classes are treated as negative ones. For the ease of calculation, both the positive and negative features are clustered into 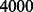 centers. After discriminative feature selection by the EM iterations, we only keep the discriminative features for the positive dataset, denoted as 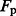, in our selection result. These features in 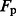 represent those that help to distinguish the images in the 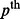 class from others. These procedures are repeated for 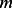 times and the final codewords are clustered with all the features in 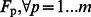.

To verify the results, three datasets discussed above are all included in the experiment. In the SAR dataset, 10 images per category are used as training samples and the rest are test samples. In Fifteen Scene dataset, 100 images in each category are randomly selected as training samples and the rest are for test. In Indoor Scene dataset, we use the training and test samples provided in [22]. In the simulation, the multi-class SVM is applied and we strictly follow the implementations in some previous works [20], [32] to conduct the experiments. In addition, we vary the number of codewords as 200, 400, 600, and 800 to investigate the robustness of the proposed method. Moreover, two benchmark feature coding algorithms, *i.e.* hard assignment [23] and kernel assignment [20] are respectively applied to the selected codewords for image-level feature generation. As indicated in [22], the categorization of indoor images is quite challenging and therefore we further exploit the 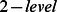 spatial-pyramid [23] to improve the accuracy on MIT Indoor Scene dataset.

**Figure 6:**
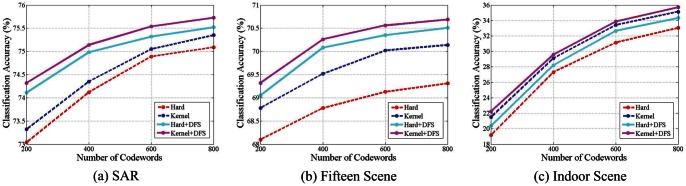
Classification accuracy on three datasets with different numbers of codeword.

Fig. 6 shows the average classification accuracy of three datasets with regard to different sizes of codebook. It is shown that with the increase of the number of codewords, the classification accuracy is correspondingly improved. As expected, by using the same codebook, kernel assignment achieves higher performance than hard assignment. In Fig. 6, solid lines refer to categorization accuracies achieved from the codebooks generated by discriminative feature selection (DFS) using our proposed model, whereas the dashed lines represent those with respect to codebooks generated by simply clustering all features from multiple classes. It is indicated that by using discriminative feature selection (DFS) to generate the codebooks, the categorization accuracies on three datasets with regard to different sizes of the codebook are apparently enhanced.

It is worth noting that on SAR dataset which has only six categories, with DFS there is a considerable improvement on classification performance. In contrast, for the challenging Indoor Scene dataset with 67 categories, the enhancement in classification is less significant. We attribute this to the difficulty in selecting discriminative features from a large number of classes. Specifically, it is hard to separate discriminative features from the common features among as many as 67 classes, while it is comparatively easy to address 6 classes. But, even on these difficult task, our DFS method still improves the classification accuracy for 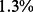 on the MIT Indoor Scene dataset with 800 codewords.

In the discussions aforementioned, we have verified the effectiveness of DFS on image categorization. Related image categorization work [23] has indicated that the classification accuracy could be significantly improved by using the pyramid methods. In order to achieve classification results comparable with other state-of-the-arts, we further follow the idea in [23] to conduct the classification with a 3-level pyramid. The number of codewords is fixed as 800. The experimental results are repeated for 10 times and the corresponding results (average accuracy 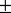 std 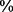) are reported in Table 3. In the indoor scene dataset, to make the result comparable with previous published paper, we directly use the training and testing list provided in [22] and the experiment is only implemented once.

**Table 3:** Recognition Accuracy of Multi-class Classification Tasks Using Spatial-Pyramid(%).

Datasets	Hard	Kernel	Hard + DFS	Kernel + DFS
SAR	81.2±0.9	82.9±0.7	84.4±0.6	87.3±0.5
Fifteen Scene	75.8±0.9	79.2±1.0	78.3±1.1	83.7±1.2
MIT Indoor Scene	34.1	36.3	37.9	38.6

In Table 3, it is indicated that by using 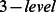 spatial-pyramid and a larger number of codewords, the classification performance is significantly enhanced. On SAR dataset, the classification accuracy can achieve higher than 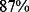. Specifically, on Fifteen Scene dataset, the classification accuracy is as high as 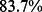, which is comparable with many state-of-the-arts. On the very challenging indoor scene dataset, we produce a categorization rate as high as 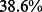 by the DFS method with soft assignment. The result is higher than some recent reported results by using the same training and testing samples [22].

## Limitations of Study

Although there is much remains to be done, our work has introduced the preliminary idea of finding image differences by a generative probabilistic model. In other words, we have shown that discovering image differences among multiple classes indeed helps recognition tasks. Although the presented studies have yielded some preliminary findings, a few limitations still exist in the current work.

First, in this paper, the discovery of image differences is fully implemented in a computational statistical framework. Our work fully relies on a data-driven manner to identify the discriminative patterns. Therefore, the learning results are partially determined by what kind of local descriptors are used as the input of the model. In this work, we choose the most prevalent image descriptor, *i.e.* the SIFT feature, to represent the contents of images. However, describing image content by a single feature will be possibly insufficient. Therefore, there exists some artifacts on the discriminative feature selection results in Fig. 4. In our future works, we may consider to find the differences among images by using multiple descriptors.

Besides, with regard to the image classification accuracy, the performance of this method is not the highest reported one on some benchmarks [19]. In previous works for image categorization, multiple implementations have been adopted to improve the classification performances. For example, multiple descriptors were used in some systems with nonlinear classifier. The final classification results are voted by different discriminative machines. Therefore, there is no surprise to find some higher accuracy in these papers. In contrast, in this paper we only used one kind of descriptors with a single SVM classifier for image categorization. Even with such a simple implementation, after spatial pyramid enhancement [23], the classification results are very competitive with previous published results that used multiple approaches for image understanding. Meanwhile, we would like to emphasize that the core contribution of this work is not in the pursuit of the highest quantity on the classification accuracy. The aim of this paper is to reveal that differences among images indeed help recognition. Accordingly, the experiments designed in this work all focus on this prominent goal.

## Conclusions

This paper proposes a generative model to interpret how different and common features are generated in images from multiple classes. The whole mathematical framework can be efficiently solved in an EM paradigm with very robust convergence. The experimental results from different perspectives, including visual effectiveness comparison, bi-class categorization and multi-class classifications, all verify that differences on images is very critical for improving the classification performances. Although we concern on the task of image categorization, the effectiveness of the proposed model is obviously beyond the scope discussed in this paper. The statistical model is a a general method to distinguish discriminative features from common ones. Therefore, it can be applied to a diversity of practical applications that involve discriminative feature selection.

## Supporting Information

File S1 **Derivations for the EM algorithm.**
(PDF)
