# Supplementary material for: Differences Help Recognition: A Probabilistic Interpretation
Source: PLoS One. 2013 Jun 3;8(6):e63385. doi: 10.1371/journal.pone.0063385 (PMC3670869; doi:10.1371/journal.pone.0063385)
Supplement: File S1 — Derivations for the EM algorithm. (PDF) [file pone.0063385.s001.pdf]

Supplementary Materials

Yue Deng, Yanyu Zhao, Yebin Liu, Qionghai Dai

Derivations for the EM algorithm

In our model, we first calculate the conditional expectation for the complete-data likelihood,

$$\begin{aligned} Q(\Gamma,\Gamma^{k-1}) &= E_{\Phi|\mathcal{D},\Gamma^k}\{\log P(\mathcal{D},\Phi|\Gamma)\} \\ &= E_{\Phi|\mathcal{D},\Gamma^k}\{\sum_{i=1}^{N}\log P(\mathcal{D}_i,\phi_i|\Gamma)\} \\ &= \sum_{i=1}^{N} E_{\phi_i|\mathcal{D}_i,\Gamma^k}\{\log P(\mathcal{D}_i,\phi_i|\Gamma)\} \end{aligned} \tag{1}$$

According to (1), the function $Q(\Gamma,\Gamma^k)$ can be expanded as,

$$\begin{aligned} &Q(\Gamma,\Gamma^k) = \textstyle\sum_i \sum_{\phi_i} \log\left(P(\phi_i)P(x_i,l_i|\phi_i)\right)P(\phi_i|\mathcal{D}_i,\Gamma^k) \\ &= \sum_{i=1}^{N}\left\{\begin{array}{l} \left[\log P(\phi_i=1)+\log\left(\sum_{t=1}^{m}\pi_t\mathcal{N}(x_i|\mu_t,\sigma_t)\frac{\exp(w^{(l_i)}x_i+b_{l_i})}{\sum_{j=1}^{m}\exp(w^{(j)}x_i+b_j)}\right)\right]\underbrace{P(\phi_i=1|\mathcal{D}_i,\Gamma^k)}_{u_i^k} \\ +\left[\log P(\phi_i=0)+\log\left(\sum_{h=1}^{c}\hat{\pi}_h\mathcal{N}(x_i|\hat{\mu}_h,\hat{\sigma}_h)\frac{1}{m}\right)\right]\underbrace{P(\phi_i=0|\mathcal{D}_i,\Gamma^k)}_{\bar{u}_i^k} \end{array}\right\} \\ &\propto \sum_{i=1}^{N} u_i^k\log\left(\sum_{t=1}^{m}\pi_t\mathcal{N}(x_i|\mu_t,\sigma_t)\right)+u_i^k\log\frac{\exp(w^{(l_i)}x_i+b_{l_i})}{\sum_{j=1}^{m}\exp(w^{(j)}x_i+b_j)}+\bar{u}_i^k\log\left(\sum_{h=1}^{c}\hat{\pi}_h\mathcal{N}(x_i|\hat{\mu}_h,\hat{\sigma}_h)\right)=\hat{Q}(\Gamma,\Gamma^k) \end{aligned} \tag{2}$$

In (2), the conditional probability of latent variables can be estimated by Bayesian rule,

$$u_i^k = P(\phi_i=1|\mathcal{D}_i,\Gamma^k) = \frac{P(\mathcal{D}_i|\phi_i=1,\Gamma^k)P(\phi_i=1)}{P(\mathcal{D}_i|\Gamma^k)} \text{ and } \bar{u}_i^k = 1-u_i^k. \tag{3}$$

We place no partial on the prior of the feature attribute and $P(\phi_i=1)=\frac{1}{2}$. Accordingly, u_i^k and $\bar{u}_i^k$ can both be easily calculated according to the current estimation of parameters Γ^k. Therefore, the following optimization for parameter estimation is obtained,

$$\begin{aligned} \Gamma^{k+1} = \arg\max_{\Gamma}\hat{Q}(\Gamma,\Gamma^k) \\ s.t. \sum_{t=1}^{m}\pi_t = 1 \\ \sum_{h=1}^{c}\hat{\pi}_h = 1. \end{aligned} \tag{4}$$

The constraints in (4) are added to encourage the normalization on the mixture coefficients. The problem in (4) is well solved via a Lagrangian Multiplier and we give the updating rules for each parameter as follows.

$$\begin{aligned} &\pi_t = \frac{\sum_{i=1}^{N}u_j\mathbb{Z}_{it}}{\sum_{i=1}^{N}u_i^k},\ \mu_t = \frac{\sum_{i=1}^{N}u_i^k x_i\mathbb{Z}_{it}}{\sum_{i=1}^{N}u_i^k\mathbb{Z}_{it}},\ \sigma_t = \frac{\sum_{i=1}^{N}u_i^k\mathbb{Z}_{it}(x_i-\mu_t)^T(x_i-\mu_t)}{\sum_{i=1}^{N}u_i^k\mathbb{Z}_{it}}, \\ &\text{with}\quad \mathbb{Z}_{it} = \frac{\mathcal{N}(x_i|\mu_t,\sigma_t)}{\sum_{j=1}^{m}\mathcal{N}(x_i|\mu_j,\sigma_j)}. \end{aligned} \tag{5}$$

The above equations are also applied to estimate $(\hat{\pi}_h, \hat{\mu}_h, \hat{\sigma}_h)$ and we omit them here. Finally, we show how to update the parameter for the logistic regression which explicitly relies on the following optimization,

$$(w^{k+1}, b^{k+1}) = \arg\max \sum_{i=1}^{N} u_i^k \log\left(\frac{\exp(w^{(l_i)} x_i + b_{l_i})}{\sum_{j=1}^{m} \exp(w^{(j)} x_i + b_j)}\right) \tag{6}$$

The formulation in (6) resembles the typical expression of Logistic regression. The only difference is that, in our formulation, the weight u_i^k is placed before each term of the likelihood of the logistic loss. Therefore, we use the public "minFunc" optimization package in "http://www.cs.ubc.ca/ schmidtm/Software/ minFunc.html." to solve the nonlinear programming in (6).
